# *In vivo* genotoxicity assessment of a multiwalled carbon nanotube in a mouse *ex vivo* culture

**DOI:** 10.1186/s41021-022-00253-2

**Published:** 2022-10-19

**Authors:** Katsuyoshi Horibata, Hironao Takasawa, Motoki Hojo, Yuhji Taquahashi, Miyuki Shigano, Satoshi Yokota, Norihiro Kobayashi, Kei-ichi Sugiyama, Masamitsu Honma, Shuichi Hamada

**Affiliations:** 1 Division of Genetics and Mutagenesis, National Institute of Health Sciences, 3-25-26 Tonomachi, Kawasaki-ku, Kawasaki-shi, Kanagawa, 210-9501 Japan; 2 LSIM Safety Institute Corporation, 14-1 Sunayama, Kamisu-shi, Ibaraki, 314-0255 Japan; 3 Department of Pharmaceutical and Environmental Sciences, Tokyo Metropolitan Institute of Public Health, 3-24-1 Hyakunincho, Shinjuku, Tokyo 169-0073 Japan; 4 Division of Cellular and Molecular Toxicology, National Institute of Health Sciences, 3-25-26 Tonomachi, Kawasaki-ku, Kawasaki-shi, Kanagawa, 210-9501 Japan; 5 Division of Environmental Chemistry, National Institute of Health Sciences, 3-25-26 Tonomachi, Kawasaki-ku, Kawasaki-shi, Kanagawa, 210-9501 Japan; 6 Division of General Affairs, National Institute of Health Sciences, 3-25-26 Tonomachi, Kawasaki-ku, Kawasaki-shi, Kanagawa, 210-9501 Japan; 7 BoZo Research Center Inc, 1-3-11 Hanegi, Setagaya-ku, Tokyo, 156-0042 Japan

**Keywords:** Carbon nanoparticle, *In vivo* genotoxicity, Multiwalled carbon nanotubes, Lung

## Abstract

**Background:**

Multiwalled carbon nanotubes (MWCNTs) are suspected lung carcinogens because their shape and size are similar to asbestos. Various MWCNT types are manufactured; however, only MWNT-7 is classified into Group 2B by The International Agency for Research on Cancer. MWNT-7’s carcinogenicity is strongly related to inflammatory reactions. On the other hand, inconsistent results on MWNT-7 genotoxicity have been reported. We previously observed no significant differences in both *Pig-a* (blood) and *gpt* (lung) mutant frequencies between MWNT-7-intratracheally treated and negative control rats. In this study, to investigate *in vivo* MWNT-7 genotoxicity on various endpoints, we attempted to develop a lung micronucleus assay through *ex vivo* culture targeting the cellular fraction of Clara cells and alveolar Type II (AT-II) cells, known as the initiating cells of lung cancer. Using this system, we analyzed the *in vivo* MWNT-7 genotoxicity induced by both whole-body inhalation exposure and intratracheal instillation. We also conducted an erythrocyte micronucleus assay using the samples obtained from animals under intratracheal instillation to investigate the tissue specificity of MWNT-7 induced genotoxicities.

**Results:**

We detected a significant increase in the incidence of micronucleated cells derived from the cellular fraction of Clara cells and AT-II cells in both MWNT-7-treated and positive control groups compared to the negative control group under both whole-body inhalation exposures and intratracheal instillation. Additionally, the erythrocyte micronucleus assay detected a significant increase in the incidence of micronucleated reticulocytes only in the positive control group.

**Conclusions:**

Our findings indicated that MWNT-7 was genotoxic in the lungs directly exposed by both the body inhalation and intratracheal instillation but not in the hematopoietic tissue.

## Background

Fibrous or rod-shaped micrometer length particles, which share dimensions with asbestos, are carcinogenic [1–5]. Since the properties of multiwalled carbon nanotubes (MWCNTs) are similar to asbestos, MWCNTs are suspected as carcinogenic in the lung. Although various types of MWCNTs with different particle sizes and specific surface area are manufactured, only MWNT-7 (although shown as “MWCNT-7” in the reference [6], “MWNT-7” is correct), has been classified as “possibly carcinogenic to human (Group 2B)” by The International Agency for Research on Cancer (IARC) [6].

There are several conflicting reports on the *in vivo* genotoxicity of MWNT-7 (Table 1). Intratracheal MWNT-7 instillations were genotoxic to mice as demonstrated by comet assays using lung cells [7] and bronchoalveolar lavage (BAL) cells [8]. Both single pharyngeal aspiration and inhalation exposure with MWNT-7 were genotoxic to mice in comet assays using lung cells and BAL cells [9]. Interestingly, the inhalation exposure of MWNT-7 induced a higher incidence of micronucleated cells on alveolar Type II (AT-II) cells than the negative control, which supposedly represent the target cells of lung cell carcinogenesis [9]. On the other hand, *lacZ* mutant frequency analysis using lung samples collected from female Muta^TM^Mouse 60 days after the final treatment revealed that intratracheal MWNT-7 instillations were not genotoxic [10]. Additionally, we had previously reported that MWNT-7 administered intratracheally to male rats was negative in both the *Pig-a* assay using blood and the *gpt* assay using lungs collected 28 days after the single treatment [11].

**Table 1:** Summary of *in vivo* genotoxicities and experimental conditions in MWNT-7-studies

**Results**	**Exposure route**	**Genotoxicity test(s)**	**Tissue**	**Species**	**Treatment Regime**^e^	**Timings of Dissection**	**References**
Positive	whole-body inhalation^a^	*ex vivo* MN assay^b^	lung	mice	2 mg/m^3^, 2 h/d, 5 d	5 days after the final treatment	in this study
	intratracheal instillation	*ex vivo* MN assay^b^	lung	mice	0.05 mg/animal	24 h after the single treatment	in this study
	inhalation exposure	*ex vivo* MN assay	lung	mice	10.8 mg/m^3^, 4 h/d, 4 d	72 h after the inhalation exposure	[9]
	inhalation exposure	comet assay	lung	mice	8.2 mg/m^3^, 4 h/d, 4 d	24 h after the inhalation exposure	[9]
	pharyngeal instillation	comet assay	lung	mice	<200 μg/mouse	24 h after the single pharyngeal aspiration	[9]
	intratracheal instillation	comet assay	lung	mice	<162 μg/mouse	1 day after the single treatment	[8]
	intratracheal instillation	comet assay	lung	mice	<0.2 mg/animal	3h after the single administration	[7]
	intratracheal instillation	*gpt* assay^c^	lung	mice	<0.2 mg/animal/week, 4 w	8–12 weeks after the administrations	[7]
Negative	intratracheal instillation	*lacZ* assay^d^	lung	mice	<109 μg/mouse/week, 4 w	60 days after the final treatment	[10]
	intratracheal instillation	comet assay	lung	mice	<109 μg/mouse/week, 4 w	60 days after the final treatment	[10]
	intratracheal instillation	*gpt* assay^c^	lung	rats	<1 mg/kg	4 weeks after the single administration	[11]
	intratracheal instillation	*Pig-a* assay	eryth.^f^	rats	<1 mg/kg	4 weeks after the single administration	[11]
	intratracheal instillation	MN test	eryth.^f^	mice	0.05 mg/animal	24h after the single treatment	in this study
	oral gavage	MN test	b.m.^g^	mice	<20 mg/kg/d, 2d	24 h after the final administration	[12]
	inhalation exposure	γ-H2AX	eryth.^f^, lung	mice	8.2 mg/m^3^, 4 h/d, 4 d	24 h after the exposure	[9]
	inhalation exposure	MN test	b.m.^g^	mice	8.2 mg/m^3^, 4 h/d, 4 d	24 h after the exposure	[9]

^a^whole-body inhalation exposure of well-dispersed aerosol of Taquann-treated MWCNTs (T-CNTs) [13].

^b^the ex vivo micronucleus (MN) assay was performed according to the method described by Lindberg HK, et al. [14].

^c^ transgenic rodent gene mutation assay using *gpt*-delta transgenic animals

^d^transgenic rodent gene mutation assay using MutaTM mice

^e^maximum dose in each report shown with “<”

^f^ *eryth.* erythrocytes

^g^ *b.m.* bone marrow

These discrepancies on MWNT-7 genotoxicity prompted us to examine *in vivo* genotoxicity at different endpoints from our previous study. Here, we attempted to develop a lung micronucleus assay through *ex vivo* cell culture to increase cell division targeting the cellular fraction of Clara cells and AT-II cells, known as the primordial cells of lung cancer [9, 14]. Using this test system, we analyzed MWNT-7 *in vivo* genotoxicity induced by both whole-body inhalation exposure and intratracheal instillation. Additionally, we conducted an erythrocyte micronucleus assay under intratracheal instillation to investigate the tissue specificity of MWNT-7-induced genotoxicity.

## Results

### Under whole-body inhalation exposure

We conducted the first experiment with a small number of mice per dose group, followed by the confirmatory studies with more mice per dose group. Ethyl methane sulfonate (EMS) was intraperitoneally administered as a positive control.

**Fig. 1:**
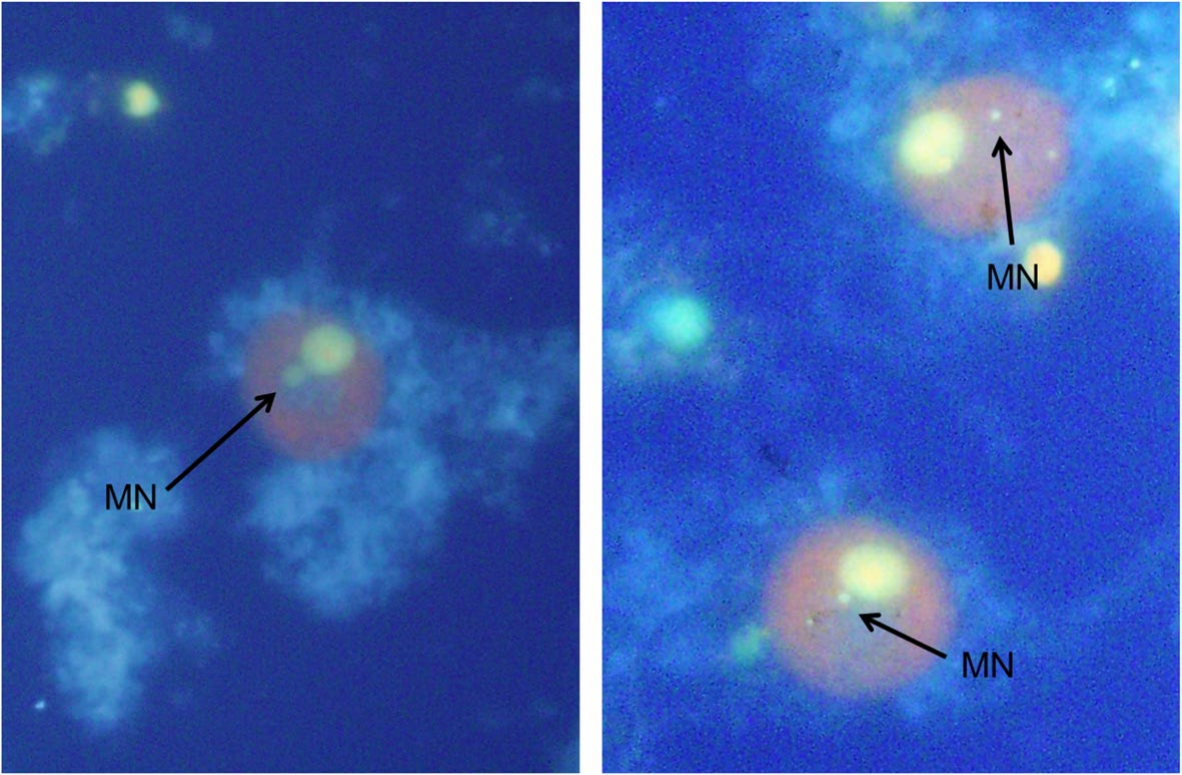
Examples of typical micronucleated cells in the MWNT-7-treated group. The prepared slide specimens were stained with a mixture of acridine orange (AO, 500 µg/ mL) -4′, 6-Diamidino-2-phenylindole (DAPI, 10 µg/ mL). The specimens were observed under a fluorescent microscope at 400× magnification with U-excitation (ultraviolet ray excitation, wave length: 330–385 nm). Micronucleated (MN) cells are shown by arrows identified as “MN.”

**Fig. 2:**
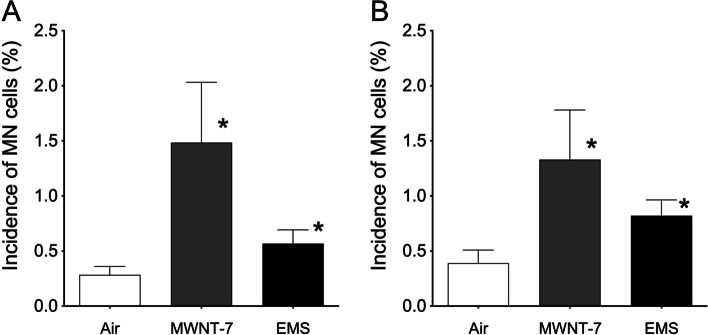
Results of the lung micronucleus assay through *ex vivo* culture after whole-body inhalation exposure. Left (**A**) and right (**B**) panels show the results of the first and confirmatory tests, respectively. The Y-axis indicates the incidence of micronucleated (MN) cells. Each treatment group is shown under the bars. The data correspond to the mean ± SD. * *p*< 0.05 compared to the control (Fisher’s exact test).

Typical micronucleated cells induced by *ex vivo* culture are shown in Fig. 1. In the first test using a small number of mice per dose group, the incidence of micronucleated cells increased significantly in the MWNT-7- and EMS-treated groups compared with the negative control [mean ± SD (%) for mice treated with air, 0.283 ± 0.076; MWNT-7, 1.483 ± 0.548; EMS, 0.567 ± 0.126] (Fig. 2A). In the confirmatory test, the incidence of micronucleated cells also increased significantly in the MWNT-7- and EMS-treated groups [mean ± SD (%) for mice treated with air, 0.390 ± 0.119; MWNT-7, 1.330 ± 0.449; EMS, 0.820 ± 0.114] (Fig. 2B).

### Under intratracheal instillation

Since we detected clear positive results in the lung micronucleus assay under whole-body inhalation exposure, we conducted the experiments under intratracheal instillation to investigate genotoxic effects caused by the different exposure methods to lung. Additionally, we conducted an erythrocyte micronucleus test to analyze the tissue specificity of MWNT-7-induced genotoxicity.

**Fig. 3:**
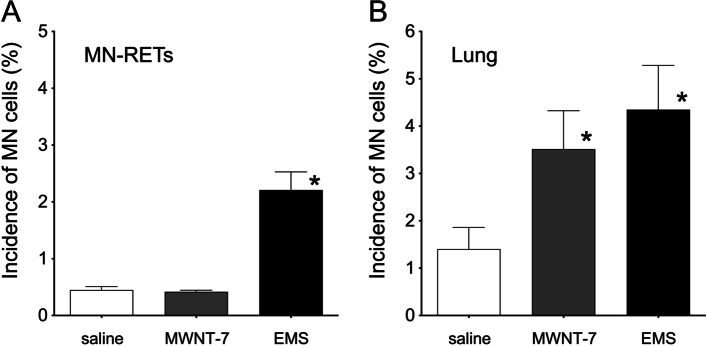
Results of the erythrocyte (**A**) and the lung (**B**) micronucleus assays through *ex vivo* culture after intratracheal instillation. The Y-axis indicates the incidence of micronucleated (MN) cells. Each treatment group is shown under the bars. The data correspond to the mean ± SD. * *p*< 0.05 compared to the control (Fisher’s exact test).

Although the erythrocyte micronucleus assay detected a significant increase in the incidence of micronucleated reticulocytes only in the positive control group [mean ± SD (%) for mice treated with only vehicle, 0.448 ± 0.060; MWNT-7, 0.418 ± 0.026; EMS, 2.210 ± 0.319] (Fig. 3A), significant increases in the incidence of lung micronucleated cells in both MWNT-7 treated and positive control groups were detected [mean ± SD (%) for mice treated with only vehicle, 1.403 ± 0.457; MWNT-7, 3.517 ± 0.808; EMS, 4.348 ± 0.936] (Fig. 3B).

## Discussion

MWCNTs are classified based on its characteristic size and surface areas. Among these groups, only MWNT-7 has been categorized as “possibly carcinogenic to humans (Group 2B)” by the IARC based on various evidence [4–6, 15–17]. Recently, Moller et al. reviewed the genotoxicity of MWCNTs including MWNT-7 with conflicting results with respect to MWNT-7 *in vivo* genotoxicity [18]. We here summarized these conflicting results and experimental conditions in detail (Table 1).

According to several reports, comet assays targeting the lungs were positive in all but one case [7–10]. In these positive cases, the lungs were collected shortly after MWNT-7 treatment, regardless of the exposure route e.g., inhalation exposure, pharyngeal instillation, or intratracheal instillation (Table 1) [7–9]. We here attempted to develop a lung micronucleus assay through *ex vivo* culture targeting the cellular fraction of Clara cells and AT-II cells [9, 14]; we found positive results under both whole-body inhalation exposure and intratracheal instillation (Fig. 2 and 3). In the present case, we collected the lungs 5 or 2 days after whole-body inhalation exposure or intratracheal instillation, respectively.

On the other hand, in the negative case in the comet assay and the negative *lacZ* mutant frequency analysis, the lungs were collected 60 days after the final treatment [10]. We had previously reported that MWNT-7 administered intratracheally to male rats was negative in the *gpt* assay using lungs collected 28 days after a single treatment [11]. From these indications, in analyses targeting the lung, except one case in the *gpt* assay [7], it seems that MWNT-7 genotoxicity was negative when sampling times were much later than the final administration e.g., 28–60 days, but positive otherwise e.g., 3 h–5 days. Therefore, we suspected that this time difference may have influenced these results, rather than differences in exposure routes.

MWNT-7 inhalation exposure was negative in the micronucleus test using bone marrow [9]. Additionally, the standard erythrocyte micronucleus test revealed no genotoxicity of oral MWNT-7 gavage [12]. We reported a negative result in the *Pig-a* assay using blood samples collected 28 days after a single intratracheal treatment on male rats [11]. Here, we showed negative MWNT-7 genotoxicity in the erythrocyte micronucleus test (Fig. 3A). From these findings, it appears that the genotoxic effects of MWNT-7 are not systemic.

The carcinogenicity and toxicity induced by exposure to nanomaterials are strongly related to inflammatory reactions and reactive oxygen species [19–23]. Additionally, in case of MWCNTs, these effects are related to MWCNT dose, time, and physicochemical properties [24]. Interestingly, the incidence of lung micronucleated cells in the negative control group under intratracheal instillation was slightly higher than under whole-body inhalation exposure (Fig. 2 and 3B). Only air was supplied to the negative control group under whole-body inhalation exposure whereas saline containing 0.1% Tween-80 was used to treat the negative control group under intratracheal instillation. Therefore, a slight inflammatory reaction may have occurred under intratracheal instillation which slightly increased the incidence of lung micronucleated cells observed under these conditions.

All these studies indicate that exposure conditions, method, route, sampling time, and endpoints are important when evaluating MWCNT genotoxicity. Here, we showed that MWNT-7 was genotoxic in lungs directly exposed by both whole-body inhalation exposure and intratracheal instillation, although MWNT-7 was not genotoxic in the hematopoietic tissue. From our results, we still cannot explain the mechanism(s) of MWNT-7-induced *in vivo* genotoxicity; however, it is strongly suspected that this mechanism(s) is correlated with the local response at the tissue directly exposed to MWNT-7.

## Conclusions

We developed a lung micronucleus assay through *ex vivo* cell culture targeting on cellular fraction of Clara cells and AT-II, and analyzed MWNT-7 genotoxicity under whole-body inhalation and intratracheal instillation. Additionally, we conducted an erythrocyte micronucleus assay under intratracheal instillation. With respect to MWNT-7 genotoxicity, we detected a positive result in the lung micronucleus assay. Our findings indicated that MWNT-7 was genotoxic in the lungs directly exposed by both the body inhalation and intratracheal instillation, but not in the hematopoietic tissue.

## Methods

### Animals

C57BL/6NcrSlc male mice were obtained from Japan SLC (Shizuoka, Japan). Administrations were started in mice at 12-weeks-old. The animals were housed individually under specific pathogen-free conditions with a 12-h light–dark cycle. Food (CRF-1 pellet feed, Oriental Yeast Co., Ltd., Tokyo, Japan) and water were available *ad libitum*. Animal experiments were conducted in accordance with the regulations of the Animal Care and Use Committees of the National Institute of Health Sciences (approval protocol No. 693, 730, and 763).

### Test chemicals

MWNT-7 (Lot No. 060125-01k, Mitsui & Co., Ltd., Ibaraki, Japan) was prepared as described previously [4, 5, 11, 25]. Accordingly, MWNT-7 fibers are approximately 100 nm in diameter, and 27.5% of the MWCNTs are ≥5 μm in length. MWNT-7 was treated by the Taquann method to obtain well-dispersed MWNT-7 without aggregation/agglomeration [13]. EMS (Sigma-Aldrich Corporation) was dissolved in physiological saline at 2.5 and 20 mg/mL.

### Dose levels and treatments for whole-body inhalation exposure

Three or five male mice per treatment group were used for the first or confirmatory tests, respectively. The dose and administration period that had been confirmed to provide sufficient lung exposure to MWNT-7 in the previous report were applied in this study [13]. For MWNT-7-treated groups, mice were exposed to MWNT-7 at 2 mg/m^3^ for 2 h/day for five consecutive days, with systemic inhalation using Taquann Direct-Injection Whole Body Inhalation System version 2.0 [13]. For the negative control groups, mice were exposed to air in an analogous manner. EMS was intraperitoneally administered at 25 mg/kg/day at 24-h intervals for 5 consecutive days as positive controls. At 5 days after the final dosing, the animals were exsanguinated via the abdominal aorta under anesthesia and the lungs were sampled.

### Dose levels and treatments for intratracheal instillation

Six male mice per treatment group were used. A MWNT-7 suspension was prepared as described previously [26]. The dosing conditions to provide sufficient lung exposure to MWNT-7 were determined according to the previous reports, with some modifications [7, 26]. Briefly, Taquann method-treated MWNT-7 was dispersed into a physiological saline solution containing 0.1% Tween-80 at 0.5 mg/mL, and sonicated using an ultrasound bath for 30 min immediately before administration. Next, mice were intratracheally injected with vehicle (saline containing 0.1% Tween-80) or 0.05 mg/animal weight of MWNT-7. EMS was intraperitoneally administered at 200 mg/kg body weight/day at 24-h intervals for two consecutive days as positive controls. At 48 h (vehicle or MWNT-7 treated groups) or 24 h (EMS-treated group) after the final dosing, the animals were exsanguinated via the abdominal aorta under anesthesia and the blood and lungs were sampled.

### Lung micronucleus assay through *ex vivo* culture

The lung micronucleus assay through *ex vivo* culture was performed as previously described with some modifications [9, 14]. Briefly, the cellular fraction of Clara cells and AT-II cells was isolated by centrifugation in a Percoll gradient, and the isolated cells were incubated at 37˚C for 48 h. After culturing, cells were collected and treated with 1% sodium citrate solution for hypotonic treatment. Hypotonic treated cells were fixed with an acetic acid-ethanol (1: 3) fixation solution to prepare slide specimens. These specimens were stained with a mixture of acridine orange (AO, 500 µg/mL) -4′,6-diamidino-2-phenylindole (DAPI, 10 µg/mL), and observed under a fluorescent microscope at 400× magnification with U-excitation (ultraviolet ray excitation, wavelength: 330–385 nm). Relatively large round to oval cells were used as target cells (Clara cells and AT-II cells), and cells that could be identified as leukocytes were excluded from the observation target. About 2,000 cells/animal or 1,000 cells/animal from each treatment group of the whole-body inhalation exposure or intratracheal instillation, respectively, were counted and the incidence of lung micronucleated cells was calculated. Differences in the incidence of micronucleated cells in treatment groups vs. negative controls were analyzed by 2-tailed Fisher’s exact test.

### Erythrocyte micronucleus test

The erythrocyte micronucleus test was performed using the MicroFlow Plus Kit (Litron) according to manufacturer’s instructions. Briefly, blood samples were fixed with methanol below -80˚C. The samples were stained with anti-CD61 antibody (staining platelets), anti-CD71 antibody (staining reticulocytes), and/or DNA staining solution, respectively. The incidence of micronucleated reticulocytes was calculated by measuring about 20,000 reticulocytes/animal using a flow cytometer. Differences in the incidence of micronucleated cells vs. negative control groups were analyzed by 2-tailed Fisher’s exact test.

## Data Availability

All data generated or analyzed during this study are included in this published article.

## Abbreviations

MWCNT: multiwalled carbon nanotube
AT-II: alveolar Type II
EMS: ethyl methane sulfonate
AO: acridine orange
DAPI: 4′, 6-Diamidino-2-phenylindole
U-excitation: ultraviolet ray excitation

## References

[1] World Health Organization G. Asbestos and other natural mineral fibres. Environ Health Criteria. 1986;53.

[2] World Health Organization G. Chrysotile Asbestos. Environ Health Criteria. 1998;203.

[3] Hei TK, Xu A, Huang SX, Zhao Y (2006). Mechanism of fiber carcinogenesis: from reactive radical species to silencing of the beta igH3 gene. Inhalation Toxicol.

[4] Takagi A, Hirose A, Nishimura T, Fukumori N, Ogata A, Ohashi N, Kitajima S, Kanno J (2008). Induction of mesothelioma in p53+/- mouse by intraperitoneal application of multi-wall carbon nanotube. J Toxicol Sci.

[5] Sakamoto Y, Nakae D, Fukumori N, Tayama K, Maekawa A, Imai K, Hirose A, Nishimura T, Ohashi N, Ogata A (2009). Induction of mesothelioma by a single intrascrotal administration of multi-wall carbon nanotube in intact male Fischer 344 rats. J Toxicol Sci.

[6] IARC (2017). IARC monographs on the evaluation of carcinogenic risk to humans. Some nanomaterials and some fibres.

[7] Kato T, Totsuka Y, Ishino K, Matsumoto Y, Tada Y, Nakae D, Goto S, Masuda S, Ogo S, Kawanishi M (2013). Genotoxicity of multi-walled carbon nanotubes in both in vitro and *in vivo* assay systems. Nanotoxicology.

[8] Di Ianni E, Erdem JS, Moller P, Sahlgren NM, Poulsen SS, Knudsen KB, Zienolddiny S, Saber AT, Wallin H, Vogel U (2021). In vitro-*in vivo* correlations of pulmonary inflammogenicity and genotoxicity of MWCNT. Part Fibre Toxicol.

[9] Catalan J, Siivola KM, Nymark P, Lindberg H, Suhonen S, Jarventaus H, Koivisto AJ, Moreno C, Vanhala E, Wolff H (2016). In vitro and *in vivo* genotoxic effects of straight versus tangled multi-walled carbon nanotubes. Nanotoxicology.

[10] Rahman L, Jacobsen NR, Aziz SA, Wu D, Williams A, Yauk CL, White P, Wallin H, Vogel U, Halappanavar S (2017). Multi-walled carbon nanotube-induced genotoxic, inflammatory and pro-fibrotic responses in mice: Investigating the mechanisms of pulmonary carcinogenesis. Mutat Res Genet Toxicol Environ Mutagen.

[11] Horibata K, Ukai A, Ogata A, Nakae D, Ando H, Kubo Y, Nagasawa A, Yuzawa K, Honma M (2017). Absence of *in vivo* mutagenicity of multi-walled carbon nanotubes in single intratracheal instillation study using F344 gpt delta rats. Genes Environ.

[12] Ema M, Imamura T, Suzuki H, Kobayashi N, Naya M, Nakanishi J (2012). Evaluation of genotoxicity of multi-walled carbon nanotubes in a battery of in vitro and *in vivo* assays. Regul Toxicol Pharmacol.

[13] Taquahashi Y, Ogawa Y, Takagi A, Tsuji M, Morita K, Kanno J (2013). Improved dispersion method of multi-wall carbon nanotube for inhalation toxicity studies of experimental animals. J Toxicol Sci.

[14] Lindberg HK, Falck GC, Catalan J, Santonen T, Norppa H (2010). Micronucleus assay for mouse alveolar Type II and Clara cells. Environ Mol Mutagen.

[15] Nagai H, Okazaki Y, Chew SH, Misawa N, Yamashita Y, Akatsuka S, Ishihara T, Yamashita K, Yoshikawa Y, Yasui H (2011). Diameter and rigidity of multiwalled carbon nanotubes are critical factors in mesothelial injury and carcinogenesis. Proc Natl Acad Sci U S A.

[16] Takagi A, Hirose A, Futakuchi M, Tsuda H, Kanno J (2012). Dose-dependent mesothelioma induction by intraperitoneal administration of multi-wall carbon nanotubes in p53 heterozygous mice. Cancer Sci.

[17] Sargent LM, Porter DW, Staska LM, Hubbs AF, Lowry DT, Battelli L, Siegrist KJ, Kashon ML, Mercer RR, Bauer AK (2014). Promotion of lung adenocarcinoma following inhalation exposure to multi-walled carbon nanotubes. Part Fibre Toxicol.

[18] Moller P, Wils RS, Di Ianni E, Gutierrez CAT, Roursgaard M, Jacobsen NR (2021). Genotoxicity of multi-walled carbon nanotube reference materials in mammalian cells and animals. Mutat Res Rev Mutat Res.

[19] Oberdorster G, Oberdorster E, Oberdorster J (2005). Nanotoxicology: an emerging discipline evolving from studies of ultrafine particles. Environ Health Perspect.

[20] Nel A, Xia T, Madler L, Li N (2006). Toxic potential of materials at the nanolevel. Science.

[21] Xu A, Chai Y, Nohmi T, Hei TK (2009). Genotoxic responses to titanium dioxide nanoparticles and fullerene in gpt delta transgenic MEF cells. Part Fibre Toxicol.

[22] Totsuka Y, Higuchi T, Imai T, Nishikawa A, Nohmi T, Kato T, Masuda S, Kinae N, Hiyoshi K, Ogo S (2009). Genotoxicity of nano/microparticles in in vitro micronuclei, *in vivo* comet and mutation assay systems. Part Fibre Toxicol.

[23] Folkmann JK, Risom L, Jacobsen NR, Wallin H, Loft S, Moller P (2009). Oxidatively damaged DNA in rats exposed by oral gavage to C60 fullerenes and single-walled carbon nanotubes. Environ Health Perspect.

[24] Poulsen SS, Jackson P, Kling K, Knudsen KB, Skaug V, Kyjovska ZO, Thomsen BL, Clausen PA, Atluri R, Berthing T (2016). Multi-walled carbon nanotube physicochemical properties predict pulmonary inflammation and genotoxicity. Nanotoxicology.

[25] Yasui M, Kamoshita N, Nishimura T, Honma M (2015). Mechanism of induction of binucleated cells by multiwalled carbon nanotubes as revealed by live-cell imaging analysis. Genes Environ.

[26] Hojo M, Yamamoto Y, Sakamoto Y, Maeno A, Ohnuki A, Suzuki J, Inomata A, Moriyasu T, Taquahashi Y, Kanno J (2021). Histological sequence of the development of rat mesothelioma by MWCNT, with the involvement of apolipoproteins. Cancer Sci.

